# Commonalities of *Mycobacterium tuberculosis* Transcriptomes in Response to Defined Persisting Macrophage Stresses

**DOI:** 10.3389/fimmu.2022.909904

**Published:** 2022-07-01

**Authors:** Catherine Vilchèze, Bo Yan, Rosalyn Casey, Suzie Hingley-Wilson, Laurence Ettwiller, William R. Jacobs

**Affiliations:** ^1^Department of Microbiology and Immunology, Albert Einstein College of Medicine, Bronx, NY, United States; ^2^Research Department, Genome Biology Division, New England Biolabs Inc., Ipswich, MA, United States; ^3^Department of Microbial Sciences, School of Biosciences and Medicine, Faculty of Health and Medical Sciences, University of Surrey, Guildford, United Kingdom

**Keywords:** RNA-seq, tuberculosis, starvation, pH, hypoxia, dormancy

## Abstract

As the goal of a bacterium is to become bacteria, evolution has imposed continued selections for gene expression. The intracellular pathogen *Mycobacterium tuberculosis*, the causative agent of tuberculosis, has adopted a fine-tuned response to survive its host’s methods to aggressively eradicate invaders. The development of microarrays and later RNA sequencing has led to a better understanding of biological processes controlling the relationship between host and pathogens. In this study, RNA-seq was performed to detail the transcriptomes of *M. tuberculosis* grown in various conditions related to stresses endured by *M. tuberculosis* during host infection and to delineate a general stress response incurring during persisting macrophage stresses. *M. tuberculosis* was subjected to long-term growth, nutrient starvation, hypoxic and acidic environments. The commonalities between these stresses point to *M. tuberculosis* maneuvering to exploit propionate metabolism for lipid synthesis or to withstand propionate toxicity whilst in the intracellular environment. While nearly all stresses led to a general shutdown of most biological processes, up-regulation of pathways involved in the synthesis of amino acids, cofactors, and lipids were observed only in hypoxic *M. tuberculosis*. This data reveals genes and gene cohorts that are specifically or exclusively induced during all of these persisting stresses. Such knowledge could be used to design novel drug targets or to define possible *M. tuberculosis* vulnerabilities for vaccine development. Furthermore, the disruption of specific functions from this gene set will enhance our understanding of the evolutionary forces that have caused the tubercle bacillus to be a highly successful pathogen.

## Introduction

*Mycobacterium tuberculosis*, the causative agent of tuberculosis (TB), is an intracellular pathogen that has infected humans for millennia. Yet, despite a vaccine, BCG, developed a century ago and a TB pharmacopeia composed of at least 10 TB drugs targeting various *M. tuberculosis* pathways, TB remains the second leading cause of death among infectious agents after COVID-19. Notably, TB had killed one third of the world population in the 19^th^ century and over a hundred million people in the 20^th^ century. Sadly, TB will most likely become again the leading cause of death among infectious diseases in 2023. To achieve the United Nations health target of ending TB epidemic by 2035, the TB community is focusing on creating better diagnostics, better vaccines, better drugs, and shorter drug treatment. There has been less than a handful of novel TB drugs approved for TB treatment in the past 50 years and the search for new drugs that could shorten chemotherapy to weeks instead of months is actively ongoing. In contrast, the only approved TB vaccine, BCG, is more than a century old and its efficacy is far below the COVID-19 vaccines that were generated within two years of the beginning of COVID-19 pandemic. The ideal TB vaccine(s) should protect against TB infection, TB disease development and TB relapse in all age groups (1). A therapeutic vaccine as an immunoadjuvant to TB treatment could also shorten drug treatment and prevent relapse. To accomplish these goals, the identification and validation of new *M. tuberculosis* antigens and new targets that affect *M. tuberculosis* viability are needed.

The intracellular lifestyle of *M. tuberculosis* requires the bacterium to encounter multiple stresses caused by the host defense mechanisms. When *M. tuberculosis* enters its host, the bacterium is most often phagocytized by alveolar macrophages (2). Upon maturation of the phagosome, *M. tuberculosis* faces various host bactericidal and environmental defenses including acidification, reactive oxygen species, generation of nitric oxide, nutrient limitation, and a low oxygen environment (3). To survive such an environment, this exemplar intracellular pathogen has developed specific responses to evade phagosome acidification, phago-lysozyme fusion, attack from reactive oxygen and nitrogen species, and deprivation of key nutrients (3, 4). Indeed, these “toxic triggers” appear to transform the bacilli into a more stress-resistant bacilli (5, 6). In this study, we performed RNA-seq to establish gene expression and to compare the transcriptomes of *M. tuberculosis* grown *in vitro* in four conditions mimicking stresses found in the host cell: nutrient starvation, dormancy, hypoxic and acidic environment in order to identify correlating genes important in each of these “triggers”.

RNA-seq of *M. tuberculosis* grown *in vitro* in specific environments or in macrophages has been extensively analyzed to study gene adaptation to short-term stress conditions (7–13). Our goal was to establish the transcriptome of *M. tuberculosis* persisting in a variety of long-term stress conditions encountered within the macrophage environment. David Sherman’s group had performed a similar study of *M. tuberculosis* cultivated under hypoxic conditions for 14 days, which resulted in the identification of the Enduring Hypoxic Response (14). Our aim in this study is to identify the enduring stress response which leads to the survival and adaptation of increased phenotypically-resistant bacteria by comparing “intracellular” conditions *ex vitro*. Starting from the same individual cultures of *M. tuberculosis* exposed to hypoxic (1% oxygen, the intracellular oxygen level found in *M. tuberculosis*-infected human macrophages (15)), acidic, nutrient starvation or stationary phase conditions for a minimum of 14 days, RNA-seq experiments enabled us to establish similarities and differences in gene expression according to the stress induced. The genes and pathways allowing for the long-term stress survival of *M. tuberculosis* could lead to the identification of much needed novel targets for drug or vaccine development to combat the “enduring” *M. tuberculosis* pandemic.

## Materials and Methods

### Bacterial Strain and Materials

*M. tuberculosis* H37Rv was obtained from Dr. Jacobs’ laboratory stock. Chemicals were obtained from Thermo Fisher Scientific (Waltham, MA) or Sigma (St Louis, MO).

### Growth Conditions for *M. tuberculosis* Cultures Under Various Stresses Prior to RNA Isolation

*M. tuberculosis* H37Rv was grown in Middlebrook 7H9 (Difco, Sparks, MD) supplemented with 10% (v/v) OADC enrichment (5% (w/v) bovine albumin fraction V, 2% (w/v) dextrose, 0.003% (w/v) catalase, 0.85% (w/v) sodium chloride in water), 0.2% (v/v) glycerol and 0.05% (v/v) tyloxapol (7H9-OADC-gly-tylo). The *M. tuberculosis* H37Rv culture was then sub-cultured into 15 individual cultures [10 ml, OD_600 nm_ ~ 0.05; three replicates per condition for the five conditions tested (exponential growth, nutrient starvation, stationary phase, hypoxic, and acidic environment)] which were incubated shaking at 37°C to the appropriate OD_600 nm_ and exposed to the different conditions. Three *M. tuberculosis* H37Rv cultures were incubated shaking at 37°C to an OD_600 nm_ ~ 0.63 for exponential phase condition. For nutrient starvation, three *M. tuberculosis* cultures grown to OD_600 nm_ ~ 0.8 were washed twice in PBS supplemented with 0.05% tyloxapol (v/v) and resuspended in 10 ml of PBS supplemented with 0.05% tyloxapol (v/v). The cultures were incubated shaking at 37°C for 14 days. Stationary phase cultures of *M. tuberculosis* were obtained by incubating three *M. tuberculosis* cultures shaking at 37°C for four weeks. Three actively growing *M. tuberculosis* cultures (10 ml, OD_600 nm_ ~ 0.15) were grown under hypoxic conditions by shifting the cultures to a glove box (Coy Laboratory Products, Grass Lake, MI) containing 1% oxygen and 5% CO_2_. The level of oxygen was maintained at 1% by automatic injection of nitrogen into the chamber. The cultures were incubated at 37°C in 1% oxygen for 14 days. Three actively growing *M. tuberculosis* cultures (10 ml, OD_600 nm_ ~ 0.1) in 7H9-OADC-gly-tylo were spun down and resuspended in 7H9-OADC-gly-tylo acidified to pH 5.8 with hydrochloric acid. The cultures were grown shaking at 37°C for three weeks, diluted to an OD_600 nm_ ~ 0.1 in 7H9-OADC-gly-tylo-pH 5.8 and incubated shaking at 37°C for 14 days. Samples from each stress conditions were taken at different times, diluted in PBS and plated onto Middlebrook 7H10 (Difco) supplemented with 10% (v/v) OADC enrichment and 0.2% (v/v) glycerol for colony-forming units (CFUs) determination.

### RNA Isolation

The 15 cultures of *M. tuberculosis* (see above) were spun down and resuspended in 1 ml Qiagen RNA protect (Qiagen, Germantown, MD) overnight. The suspensions were spun down and the cell pellets were resuspended in 1 ml TRIzol (Invitrogen). The suspensions were transferred to Fast-Prep Blue Cap tubes and processed in a Fast-Prep apparatus (MP Biomedicals, speed 6.5, 30 sec, 3 times with a 5 min rest in ice between run). The samples were spun down at 4°C for 10 min. The supernatants (0.7 ml) were removed to new tube containing 0.7 ml of 100% ethanol. The samples were then transferred to a Zymo-Spin IICR Column (Zymo Research, Irvine, CA) and processed according to the manufacturer’s Direct-zol™ RNA Miniprep protocol.

### RNAseq Library Preparation

The same amount of RNA (about 500 ng per sample) was used for library preparation of each sample. Total RNA was processed with NEBNext bacterial rRNA removal kit (E7850) with the addition of 2uM custom probes targeting *M. tuberculosis* to remove rRNA. Subsequently the rRNA depleted RNA was used to prepare directional RNAseq library using NEBNext Ultra II Directional RNA Library Prep Kit (E7760) following the manufacturer’s instructions. The dual indexed libraries were sequenced using Illumina Novaseq (50bp paired-end) to generate between 5-7 million reads.

### Bioinformatic Analysis

The genome version ASM19595v2 and corresponding annotation of *M. tuberculosis* H37Rv strain were used for all the analyses. We trimmed the illumina adapter sequence from the paired-end RNA-seq reads using Trimgalore (version 0.4.4 with parameters –length 25 –stringency 3) (https://github.com/FelixKrueger/TrimGalore). Next, we aligned the trimmed paired-end reads to the H37Rv genome using bowtie2 alignment (version 4.8.5) (16). The number of reads that were directionally aligned to the annotated genes and FPKM (Fragments Per Kilobase of transcript per Million mapped reads) were counted using featureCounts (version 1.6.0) with parameters: -p -M -t gene -g ID -s 2. RNA-seq normalization was based on similar number of reads for each library and DESeq2 (version 1.30.1) (17) in the statistical analysis, which considers the differences in library sizes (number of reads mapping to *M. tuberculosis*) and composition (gene expression). The Normalization was done before the differential gene expression analysis (DESeq2 counts with normalized=TRUE). The differential expression analysis was performed considering the replicates with the default parameters of DESeq2: the gene expression under the four stress conditions was compared to the expression under the exponential stage. The genes having padj <0.05 were defined as significantly differentially expressed genes. Expression clustering was performed on the output of DESeq2 using the log2FoldChange. Clustering was performed using pheatmap with euclidean distances, a maxclust of 35 and a complete clustering method (clustering_method = “complete”).

## Results

### Growth Conditions and RNA-Seq Experiments

Regulation of gene expression constitutes a main component of the mycobacterial response to environmental conditions and stress changes. To investigate the effects of the intracellular stresses encountered on *M. tuberculosis* gene expression, *M. tuberculosis* was grown *in vitro* in four stress conditions intending to resemble the conditions present in macrophages infected by *M. tuberculosis*: nutrient starvation (PBS), dormancy (stationary phase), acidic (pH 5.8) and hypoxic (1% oxygen) environment for 14 days. The gene expression profiles were obtained by RNA sequencing of the transcriptome. Directional RNA-seq was performed in biological duplicates after isolation of total RNA from *M. tuberculosis* grown in exponential phase or under the four stress conditions described above. Prior to library preparation, rRNA were removed and about 5-7 million paired-end illumina reads were sequenced for each library. Reads were mapped to the *M. tuberculosis* reference genome using bowtie2 alignment (16). rRNA represented less than 1% of all RNA-seq reads for most libraries (**Table S1**). The expression levels of the 4014 annotated non rRNA genes were estimated based on the number of mapped reads using DESeq2 (17). Thereby, the differentially expressed genes in each stress condition compared to the exponential growth condition were identified using Deseq2 (**Figure S1A**). Gene expression between the two biological replicates reveals high correlation (Pearson correlation > 0.99, P-value < 2.2x10^-16^) for all conditions examined (**Figure S1B**).

### Genes Differentially Regulated in All Stress Conditions

**Figure 1:**
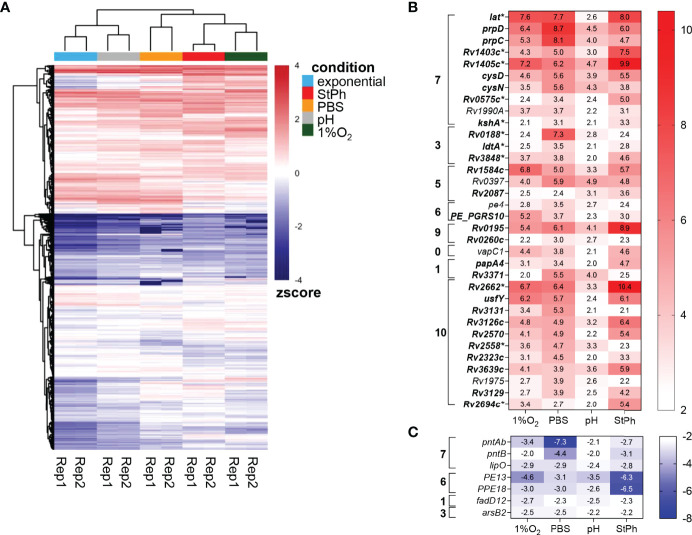
**(A)** Heat map showing the expression level of 4014 genes using Z-score for normalized FPKM value. Red represents high expression level, while blue represents low expression level. The two-way hierarchical clustering is based on euclidean distance, showing similarity between different samples. **(B, C)** Heat map of the genes up- **(B)** and down-regulated **(C)** in all stress conditions. The log2 fold change is indicated in each cell for the specific gene/stress condition combination: hypoxic, 1%O_2_; nutrient starvation, PBS; acidic, pH; stationary phase, StPh. Functional categories are as follows: 0 virulence, detoxification, adaptation; 1 lipid metabolism; 2 information pathways; 3 cell wall and cell processes; 5 insertion seqs and phages; 6 PE/PPE; 7 intermediary metabolism and respiration; 9 regulatory proteins; 10 conserved hypotheticals. *Genes part of the enduring hypoxic response.

The RNA-seq set encompassed expression data for 4014 genes with a fragment per kilobase million (FPKM) ranging from 0 to 8600 (**Table S2a**). To compare the transcriptome of *M. tuberculosis* grown under all conditions, the euclidean distance between samples was measured to describe the similarity of gene expression profile (**Figure 1**). Among the four stress conditions, the gene expression pattern of acidic condition was most similar to the exponential growth condition, while the pattern of hypoxic condition was the most different. The gene expression in each stress condition was then compared to the transcriptome of exponential phase *M. tuberculosis* grown exponentially in standard medium. Genes were considered significantly up- or down-regulated compared to exponential growth conditions when the log2 fold change in expression was higher than 2 or lower than -2, respectively, with an adjusted *p* value of less than 0.05. An average 1050 genes were differentially transcribed in each stress condition except for the acidic stress, which had less than 200 genes differentially regulated (**Table 1**). This low number of genes differentially expressed at acidic pH might be due to the ability of *M. tuberculosis* to establish a slow and partial growth at pH 5.8, while growth was arrested in the other stress conditions (**Figure S2**). The most represented functional categories for differentially expressed genes belonged to the cell wall and cell processes (17-19%), intermediary metabolism and respiration (20-25%) and conserved hypotheticals (25-29%) [based on mycobrowser functional categories (mycobrowser.epfl.ch/)]. The dataset was initially partitioned into 35 expression clusters (**Table S2b**), which confirmed that the hypoxic and stationary phase conditions had the most similarities while the acidic condition stands apart from the other three stress conditions (**Figure S3**). This expression clustering also uncovered sets of genes that were commonly differentially regulated in all stress conditions.

**Table 1:** Classification of the genes induced or repressed in *M. tuberculosis* grown under hypoxic, nutrient starvation, acidic or stationary phase condition compared to exponential growth condition.

Functional category	Hypoxic		Starvation		Acidic		Stationary phase	
log2 fold change	> 2	< -2	> 2	< -2	> 2	< -2	> 2	< -2
virulence, detoxification, adaptation	52 (22)	22 (9)	24 (10)	26 (11)	4 (2)	1 (0)	53 (23)	27 (12)
lipid metabolism	49 (20)	29 (12)	24 (10)	30 (12)	9 (4)	4 (2)	22 (9)	39 (16)
information pathways	24 (10)	31 (13)	22 (9)	36 (15)	2 (1)	3 (1)	19 (8)	33 (14)
cell wall and cell processes	113 (15)	115 (15)	90 (12)	88 (11)	19 (2)	11 (1)	79 (10)	123 (16)
insertion seqs and phages	39 (27)	6 (4)	27 (18)	2 (1)	6 (4)	3 (2)	49 (33)	3 (2)
PE/PPE	41 (24)	6 (4)	26 (15)	11 (7)	10 (6)	3 (2)	30 (18)	14 (8)
intermediary metabolism and respiration	167 (18)	103 (11)	85 (9)	167 (18)	27 (3)	17 (2)	106 (12)	110 (12)
unknown	3 (19)	1 (6)	3 (19)	1 (6)	1 (6)	0 (0)	7 (44)	1 (6)
regulatory proteins	56 (29)	14 (7)	42 (22)	12 (6)	4 (2)	1 (1)	63 (32)	10 (5)
conserved hypotheticals	152 (14)	135 (12)	194 (17)	93 (8)	46 (4)	9 (1)	205 (18)	107 (10)

In parenthesis is given the percentage relative to the total number of genes in the M. tuberculosis genome in each category.

Thirty four genes were upregulated from 4- to 1000-fold in all stress conditions (**Figure 1**) and were found in all functional categories except information pathways. The most represented functional category was intermediary metabolism and respiration (33%). Among them, the genes *prpC* and *prpD*, encoding the methylcitrate synthase and the methylcitrate dehydratase respectively, were among the most upregulated in all conditions (**Figures 1**, **S4**). These genes are part of the methylcitrate cycle and are essential for propionate metabolism in *M. tuberculosis* (18, 19). The lysine-epsilon aminotransferase encoded by *lat* (*Rv3290c*) was also highly induced (>100-fold) in all but acidic conditions. This gene was previously shown to be highly upregulated during starvation (20). Lat generates glutamate from lysine and 2-oxoglutarate. A study by Lee et al. demonstrated that *M. tuberculosis* utilizes the methylcitrate cycle and the conversion of glutamine to glutamate to counteract propionate toxicity (21). Although our data did not show any induction of the glutamate synthases GltB/D (**Table S2c**), up-regulation of Lat may result in a similar increase in glutamate to prevent propionate toxicity during various stresses. *lat* is also part of the enduring hypoxic response, a set of 229 genes responding to hypoxia over the long term (14). Eleven genes in our set of 34 genes induced in all stress conditions are part of the enduring hypoxic response (**Figure 1**, genes marked with *). Among them was *kshA*, a gene encoding the oxygenase of 3-ketosteroid 9α-hydroxylase, part of cholesterol catabolism. Cholesterol is a major food source for the bacilli when within the macrophage environment (22, 23). Degradation of the cholesterol rings involves three enzymes: the 3-ketosteroid 1Δ-dehydrogenase KstD, KshA and its reductase counterpart KshB (24). The upregulation of *kstD* and *kshB* in two to three of the stress conditions (**Table S2c**) indicates that *M. tuberculosis* is primed to catabolize cholesterol under specific stress conditions. *kshAB* genes have been found to be essential for *M. tuberculosis* virulence and growth in macrophage and in mice (25). In addition, two putative methyltransferases encoded by *Rv1403c* and *Rv1405c*, part of the enduring hypoxic response, were also induced in all the stress conditions. These two genes were previously found to increase in response to the intracellular environment, hypoxia, and acidic conditions (26–29).

The ATP sulfurylases encoded by *cysN* and *cysD*, which convert ATP to adenosine 5’-phosphosulfate (APS), were also upregulated in all stress conditions. These two genes control the first steps in sulfur metabolism in *M. tuberculosis*. APS is used for the synthesis of cysteine, proteins and redox defense (30). Phosphorylation of APS yields PAPS (3′-phosphoadenosine 5′-phosphosulfate), which is a sulfuryl donor to various metabolites and lipids such as sulfolipids, and a possible regulator of metabolic pathways (30). *cysN* and *cysD* were shown to be induced during mouse and human macrophage infection (26, 29). Schnappinger et al. had previously defined a multiple stress response encompassing *cysN, cysD* as well as *hsp, clpB, Rv2660c* and *sigB (* 29*)*, genes that were also induced in at least two of our conditions (**Table S2c**). The other genes upregulated in all stress conditions with known functions encoded the ribonuclease VapC1, the triacylglycerol synthase Rv3371, the polyketide synthase PapA4, PE4, PE_PGRS10, the L,D-transpeptidase LdtA, and three transcriptional regulatory proteins Rv0195, Rv0260c and Rv1990A. *Rv3371* was previously shown to be upregulated in hypoxia leading the authors to postulate that, under low growth, *M. tuberculosis* shuffles the flow of carbon towards fatty acid synthesis and away from energy and biosynthetic metabolisms (31). Rastogi et al. reported that *Rv3371* expression was the highest in stationary phase, under hypoxia and during nutrient starvation (32).

Only seven genes were found to be downregulated in all stress conditions (**Figure 1**). Among them were *pntAb* and *pntB*, two genes encoding NAD(P) transhydrogenases that generate NADH and NADP+ from NAD+ and NADPH, or the reversible reaction (33). *pntAa*, which encodes the third NAD(P) transhydrogenase, was also downregulated in all stress conditions, but was significant only in hypoxia and starvation conditions. These proteins are thought to help in maintaining redox balance in bacteria. The proton motive force generated in these reactions is linked to ATP synthesis (34), which matches the downregulation of genes involved in ATP biosynthesis observed in these conditions (see below). *pe13* and *ppe18* are part of the type VII secretion system ESX-5b (*Rv1195-Rv1198*), a duplication of the ESX-5 system, possibly involved in the transport of a subset of proteins secreted by ESX-5 (35). Of note, the four genes in the ESX-5b cluster were the most down-regulated genes in the stationary phase condition. Two genes, *lipO* and *fadD12*, involved in lipid metabolism/degradation with no known functions were also part of this group along with the anion transporter encoded by *arsB2.*

The expression cluster also indicated that these stress conditions differed in the way they regulated pathways involved in metabolism, cellular and information processes. We then clustered the RNA-seq data based on gene annotation using kegg pathways analysis (www.kegg.jp) and examined how these stress conditions regulated energy, central carbon, amino acid, cofactors, vitamins metabolisms as well as stress, virulence and cell wall components.

### Stress Response

**Figure 2:**
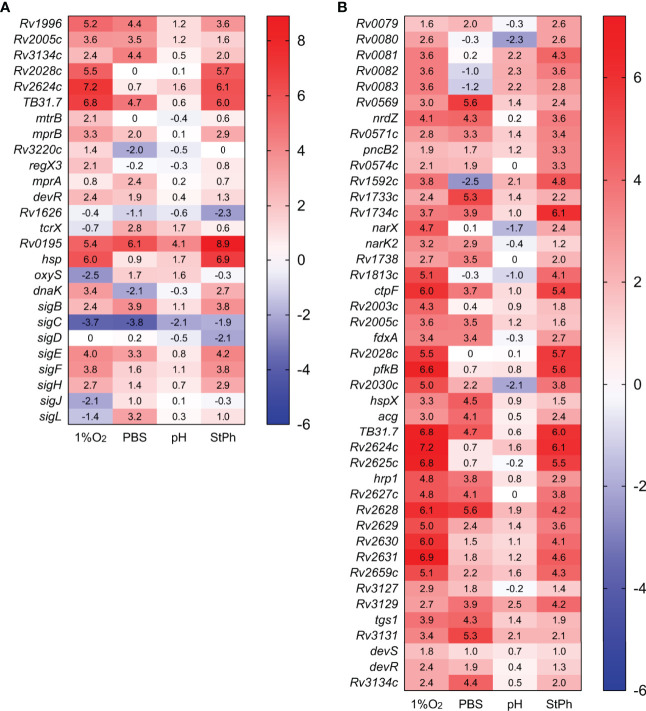
Heat map of genes differentially expressed in stress conditions **(A)** or in the dormancy regulon **(B)**. The log2 fold change for each gene and condition is indicated in the cell.

We first looked whether the conditions applied to the *M. tuberculosis* cultures did generate a stress response signature. There are eight proteins annotated as universal stress proteins (USPs) in the *M. tuberculosis* genome and six out of eight genes encoding these proteins (Rv1996, Rv2005c, Rv2028c, Rv2623, Rv2624c, Rv3134c) were upregulated in at least two of our stress conditions (**Figure 2**) and have also been determined to be upregulated when within the macrophage in several transcriptional studies (29, 36, 37). Prior studies have determined no survival defect in both murine and human macrophages or in response to any of the stresses noted here of whole gene knock-outs of *Rv1996, Rv2005c, Rv2028c* and *Rv2623* (38) generated by specialized transduction (39). Interestingly, none of the genes encoding these proteins were induced in the acidic environment, which was also noted in a prior pH-centered study (40). The *M. tuberculosis* stress response also includes two component systems. The two-component transcriptional regulatory protein encoded by *Rv0195* was induced in all stress conditions. Rv0195 belongs to the LuxR family regulator and is required for *M. tuberculosis* survival in hypoxia, recovery from dormancy, growth in macrophages and virulence in mice (41). Dormancy, which can be generated in *M. tuberculosis* cultures grown to stationary phase, under hypoxia, or starved for nutrients is also controlled by the two-component transcriptional regulatory system DevR/DevS. *devR* was slightly induced in these conditions and the DevR (also known as DosR) regulon, which is composed of up to 47 genes and includes many of the above mentioned USPs, was highly induced (**Figures 2**, **S4**).

Other members of two-components systems were induced such as *mprAB, regX3* or *tcrX* (**Figure 2**). Bretl et al. (42) had previously shown that MprA and DosR coregulated a general stress-responsive operon composed of *Rv1812c* and *Rv1813c*, two genes that are also upregulated in our hypoxic and stationary phase conditions (**Table S2c).** The mycobacterial response to various stress situations can also be triggered and controlled by sigma factors. *M. tuberculosis* has 13 sigma factors: one essential and twelve accessory sigma factors. Four sigma factors (*sigB, sigE, sigF, sigH*) were induced in at least two conditions (but not acidic), while *sigC* was repressed in all conditions, especially in hypoxia and nutrient starvation (**Figure 2**). SigE and MprAB have been proposed to constitute a network that responds to surface stress such as cell wall damages, a network that could additionally include the chaperone DnaK (43, 44), which was induced in the hypoxic and stationary phase conditions but repressed in nutrient starvation condition. The conditions tested where growth arrest was observed did indeed induce the persister (DosR) regulon and a stress response, while the acidic condition induced only a few of these genes, probably due to the slow growth of *M. tuberculosis* in this environment or successful and swift adaptation of *M. tuberculosis* to low pH.

### Energy Metabolism

**Figure 3:**
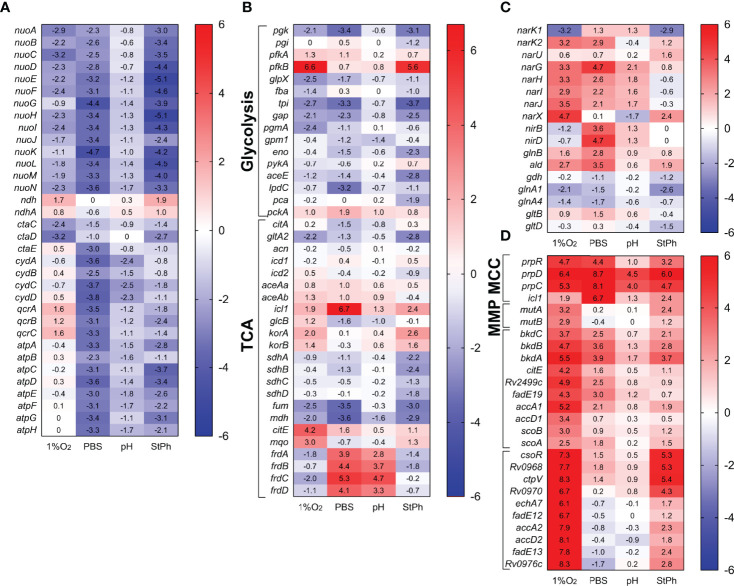
Heat map of genes differentially transcribed in **(A)** electron transfer chain, **(B)** glycolysis and tricarboxylic acid cycle (TCA), **(C)** nitrogen metabolism, **(D)** propionate metabolism (MCC, methylcitrate cycle; MMP, methylmalonate pathway). The log2 fold change for each gene and condition is indicated in the cell.

Genes that encode the type I NADH dehydrogenase (*nuoA-N*), the cytochrome c reductase (*qcrCAB*), the aa3 cytochrome c oxidase (*ctaCDE*), the cytochrome *bd* oxidase (*cydABDC*) and the ATP synthase (*atpA-H*) were repressed in most stress conditions (**Figure 3**). The most pronounced pattern was observed in nutrient starvation with high downregulation of these complexes and upregulation of genes encoding the fumarate (*frdABCD*) and nitrate (*narGHIJ*) reductases (**Figure 3**), which act as electron acceptors in anaerobic respiration. The NarGHIJ proteins reduce nitrate to nitrite, a toxic metabolite that is detoxified by the nitrite reductases NirBD, which reduce nitrite to ammonia. The nitrite reductases *nirBD* were also highly induced during nutrient starvation, but none of the genes involved in ammonia utilization for glutamine (*glnA1-4*) or glutamate (*gltBD*) biosynthesis were differently expressed (**Figure 3**). Others have suggested that nitrite produced by the Nar complex is not reduced by NirBD (45), but instead exported by NarK2 (46), which was also induced in the same conditions (**Figure 3**). The upregulation of nitrate and fumarate reductases was also observed in acidic conditions while hypoxic *M. tuberculosis* only induced the nitrate reductases. None of these reductases were differentially regulated in stationary phase. Our data support the idea that anaerobic respiration plays an important role in the survival of *M. tuberculosis* cultivated under hypoxic, acidic, or nutrient starvation conditions, but not in stationary phase *M. tuberculosis*.

### Central Carbon Metabolism

Central carbon metabolism encompasses several pathways that include glycolysis, gluconeogenesis, the pentose phosphate pathway, and the citric acid cycle with its glyoxylate shunt that provides essential components for active growth (47). As expected for conditions where *M. tuberculosis* is in a slow- or non-replicating state, the genes involved in these pathways were repressed or not induced (**Figure 3**). Furthermore, genes involved in propionate metabolism were highly induced (**Figure 3**). *prpC, prpD* and *icl1*, genes from the methylcitrate cycle (MCC), which metabolizes propionyl-CoA generated by β-oxidation of odd-chain fatty acids to produce succinate and pyruvate, were among the most expressed genes in all stress conditions (see above). While the genes of the methylcitrate cycle were upregulated in all conditions, genes belonging to the methylmalonyl pathway, which converts propionyl-CoA to succinate *via* methylmalonyl-CoA, were specifically induced in the hypoxic samples. These systems are thought to purge excess propionate, a toxic metabolite, from bacterial cells (19, 22). Others have demonstrated that *M. tuberculosis* can reverse the methylcitrate cycle when grown in glycerol and oleic acid to produce propionyl-CoA for the biosynthesis of lipids (48). The induction of *bkdABC*, genes involved in the degradation of valine, leucine and isoleucine to produce propionyl-CoA or acetyl-CoA, as well as the induction of the *accA1-accD1* operon implicated in leucine degradation (49) and therefore propionyl-CoA production could point towards this direction (**Figure 3**). The lack of induction of the TCA cycle suggests that in these stress conditions, *M. tuberculosis* rather shuttles propionyl-CoA into the synthesis of methyl-branched lipids such as phthiocerol-dimycocerosates (PDIMs) and sulfolipids (50), lipids of critical importance for *M. tuberculosis’* virulence.

### Amino Acid Biosynthesis

**Figure 4:**
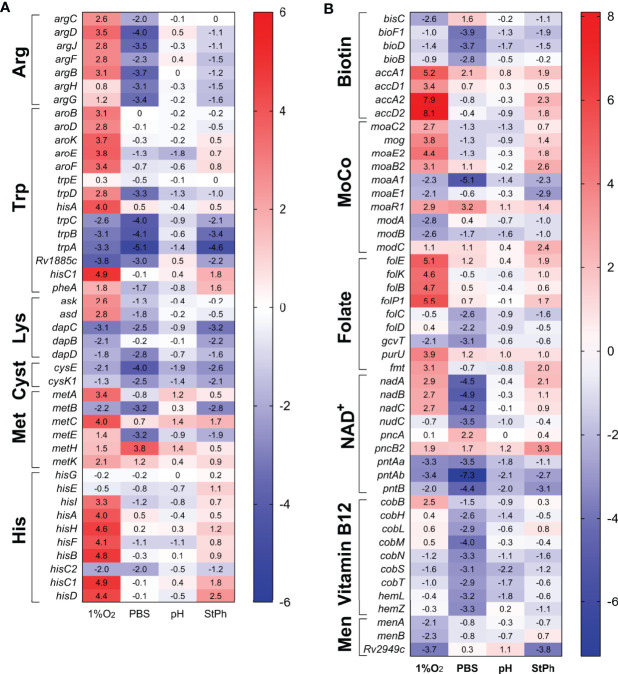
Heat map of genes differentially expressed in **(A)** amino acid, **(B)** vitamins and cofactor biosynthesis and metabolism. The log2 fold change for each gene and condition is indicated in the cell.

While the genes involved in the biosynthesis of most amino acids were neither induced nor repressed in the stress conditions, several pathways involved in the biosynthesis of arginine, aromatic amino acids, lysine, histidine and methionine were highly induced in hypoxia, while being repressed or not induced in the other conditions (**Figure 4**). Of note, six of these amino acids (histidine, lysine, methionine, phenlylalanine, threonine and tryptophan) are included in the nine essential amino acids in humans. The first steps in the formation of lysine, homocysteine or threonine comprise the aspartokinase and the aspartate dehydrogenase encoded by *ask* and *asd*, respectively. *ask* and *asd* were induced in hypoxia, but surprisingly, the other genes involved in the biosynthesis of these amino acids (*dapABCDF-lysA, thrABC*) were not induced or were repressed (**Table S2c**). A similar phenomenon was observed with the shikimate pathway, which was highly induced in hypoxic condition (**Figures 4**, **S4**). *aroGBDEKAF* of the shikimate pathway converts phosphoenolpyruvate and D-erythrose 4-phosphate, products of glycolysis and pentose cycle, to chorismate, a precursor for tryptophan, phenylalanine and tyrosine. Yet, the genes involved in the conversion of chorismate to these aromatic amino acids (*trpABCE, pheA, tyrA* and the chorismate mutase *Rv0948c*) were also not induced or were repressed (**Figure 4**; **Table S2c**). Chorismate is also involved in the synthesis of menaquinone, folate, and siderophores. Only the folate pathway was induced in hypoxia (**Figure 4**), which might suggest that this is where chorismate is channeled. The genes associated with these amino acid biosynthesis pathways were drastically differentially regulated between hypoxia and nutrient starvation or stationary phase, indicating that although these three conditions result in persistent mycobacteria, the need for specific metabolites involved in these pathways was radically different.

### Metabolism of Cofactors and Vitamins

Annotation clustering of genes involved in the metabolism of folate, biotin, nicotinamide adenine dinucleotide NAD^+^, vitamin B12 and menaquinone revealed that these pathways were differentially regulated in hypoxia and nutrient starvation, while being non-regulated in stationary phase or acidic environment (**Figure 4**). Folate metabolism plays crucial roles in several pathways including DNA synthesis, DNA repair, cell division, amino acid biosynthesis and in the formation of the methylating agent S-adenosylmethionine. *folBKPE* genes encoding proteins for 7,8-dihydropteroate synthesis were highly upregulated in hypoxia (**Figure 4**). Yet, the genes that encode the proteins for the synthesis of dihydrofolate (DHF), tetrahydolate (THF), or 5,10-methylene-THF (*folCD, dfrA, gcvT*), were not (**Table S2c**). These folate metabolites form a cycle allowing for the methylation of dUMP to form dTMP for DNA synthesis. In parallel, this cycle also synthesizes methionine from homocysteine using vitamin B12 as a cofactor. None of the genes involved in these reactions were differentially regulated except for those associated with the synthesis of vitamin B12, which were also noticeably repressed in nutrient starvation **Figure 4**). While folate biosynthesis starts with the rearranging of guanosine triphosphate (GTP) by the guanine cyclohydrolase FolE, GTP is also the initiation point for the synthesis of the molybdopterin cofactor MoCo. MoCo is an important cofactor for oxidoreductases and is necessary for a functional nitrate reductase (51, 52). While the genes encoding the nitrate reductases were upregulated in all but stationary phase conditions, the two sets of genes associated with the synthesis of MoCo, *moaA1-E1* and *moaA2-E2*, were differentially regulated in hypoxic and stationary phase conditions (*moaA1/E1* down-regulated and *moaA2-E2* upregulated) while being mostly down-regulated in acidic and nutrient-starved conditions (**Figure 4**). This set of data is contrary to the study by Levillain et al. (51), where *moaR1* and *moaA1-moaE1* were induced in hypoxic *M. tuberculosis in vitro* and *in vivo*, resulting in the further induction of the nitrate transporter NarK2 and the nitrate reductase NarG. The authors suggested that the *moaA1-E1* gene cluster was induced only when *M. tuberculosis* was in an hypoxic and nutrient-poor environment. Notably, *moaR1* encoding the hypoxia-inducible, positive transcriptional regulator of the gene cluster *moaA1-moaE1* (51, 53) was indeed upregulated in hypoxia and in nutrient-starved *M. tuberculosis.*

Another important cofactor in metabolic reactions is NAD^+^. The genes required for the formation of nicotinic acid mononucleotide, *nadABC*, were induced in hypoxic conditions but repressed in starvation conditions (**Figure 4**). The genes associated with the NAD^+^ salvage pathway (*pncA, pncB*) were slightly upregulated while the genes encoding the proteins for the phosphorylation of NAD^+^ to NADP^+^ were repressed in all conditions. Similarly, genes encoding proteins for the formation of the vitamins menaquinone (vitamin K2) and biotin (vitamin H/B7) were down-regulated **Figure 4**). Menaquinone, which biosynthesis derives from chorismate, has an essential role in *M. tuberculosis*’ electron transport chain transferring electrons from NADH dehydrogenases to terminal electron oxidases. The repression of the menaquinone biosynthesis genes in hypoxic condition might be linked to the down-regulation of genes encoding the aa_3_ cytochrome oxidases (**Figure 3**). Biotin plays an essential role in both central carbon metabolism and lipid biosynthesis (54). Biotin is a cofactor for Pca, the pyruvate carboxylase which yields oxaloacetate for the TCA cycle (55). Biotin is also a cofactor for the biotin-dependent acyl-CoA carboxylases, which produce building blocks for the synthesis of fatty acids, methylated fatty acids and metabolism of amino acids (56). Although biotin biosynthesis genes were down-regulated in starvation condition, this was not associated with a downregulation of *accA1-3* genes (**Table S2c**). Instead, *accA1* and *accA2* were highly induced in hypoxia (**Figure 3** and **S4**). The biotin-dependent acyl-CoA carboxylases are composed of two subunits: the biotin carboxylase encoded by *accA1-3* and the carboxyltransferase encoded by *accD1-6. accA1* and *accD1* are in the same operon encompassing *bkdCBA, citE, Rv2499c, fadE19*, and *scoBA* (*Rv2495c-Rv2504c*), an operon up-regulated in most stress conditions (**Figure 3**). The complex AccA1-AccD1 functions as a 3-methylcrotonyl-CoA carboxylase involved in leucine degradation (49). *accA2* and *accD2* are also part of an operon encompassing *echA7, fadE12 and fadE13* encoding fatty acid degradation proteins, an operon (*Rv0971c-Rv0976c*) highly upregulated (20-300 fold) in hypoxic and stationary phase conditions (**Figure 3**). A study in *Mycobacterium smegmatis* by Ehebauer demonstrated that the complex AccA2-AccD2 was not involved in fatty acid and mycolic acid biosynthesis, but the authors could not determine a role for this complex (49). The requirement for specific cofactors and vitamins differed significantly between the four stress conditions. *M. tuberculosis* in an acidic environment or in stationary phase did not demonstrate any specific need for these metabolites while nutrient-starved *M. tuberculosis* down-regulated most of the genes required for the synthesis of biotin, folate, vitamin B12, NAD^+^ and molybdopterin cofactor. In contrast, hypoxic *M. tuberculosis* seemed to require multiple cofactors and vitamins for its survival since genes implicated in the biosynthesis of folate, NAD^+^ and molybdopterin cofactor were upregulated.

### The Mycobacterial Cell Envelope

**Figure 5:**
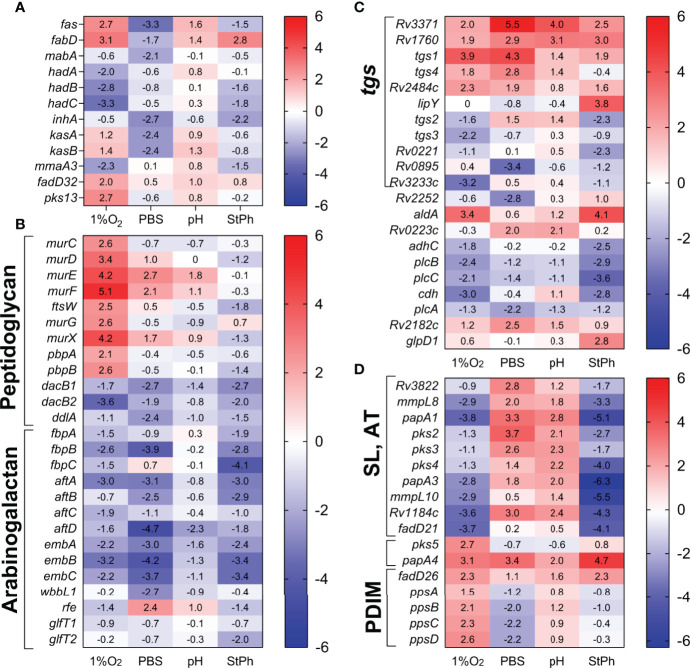
Heat map of genes differentially expressed in lipid biosynthesis and metabolism: **(A)** fatty acid and mycolic acid biosynthesis, **(B)** peptidoglycan and arabinogalactan synthesis, **(C)** glycerolipid metabolism *(tgs*, triacylglycerol synthase), **(D)** methyl-branched lipid biosynthesis [sulfolipids (SL), acylated trehaloses (AT), PDIMs]. The log2 fold change for each gene and condition is indicated in the cell.

The mycobacterial cell envelope is an intricate network of various lipids, which protects *M. tuberculosis* from any hostile aggression (host interaction during macrophage infection, antibiotic treatment, stress environment, eg intracellular) but also allows for the transport of essential nutrients and proteins. Furthermore, lipids located at the surface of the outer membrane most likely play a role in the interactions of the pathogen with its host. The cell envelope is composed of a phospholipid bilayer membrane, peptidoglycan, arabinogalactan, lipomannan, mycolic acids, lipoarabinomannan and phosphatidyl-*myo-*inositol mannosides (57). Mycolic acids, a hallmark of mycobacterium species, are long-chain α-alkyl β-hydroxy fatty acids. The fatty acid synthase type I FASI produces the α-alkyl chain. The meromycolic acids are formed by condensation of FasI fatty acids with malonyl-ACP generated from malonyl-CoA by FabD followed by elongation by the FASII system (MabA, HadABC, inhA, KasAB). Condensation of the meromycolates with the α-alkyl chain involves activation of the α-alkyl by the fatty acyl-AMP ligase FadD32 and the polyketide synthase Pks13 (57). In nutrient starvation condition, most of these systems were down-regulated while not differentially regulated in acidic growth or stationary phase (**Figure 5**). In hypoxia, *fasI*, *fabD, fadD32* and *pks13* were induced while the FASII genes were not, suggesting a need for short chain fatty acids synthesized by FASI (**Figure 5**).

The genes involved in the synthesis of glycerolipids, glycerophospholipids, arabinogalactan were repressed in most stress conditions (**Figure 5C**). *M. tuberculosis* has 16 genes annotated as possible triacylglycerol synthases (*tgs*) and 11 of them were differentially regulated in stress conditions (**Figure 5**). Triacylglycerol (TAG), a glycerol molecule with three fatty acids, is a reservoir for bacterial energy production derived from metabolism of fatty acids. One mechanism of *M. tuberculosis* survival in the host is to scavenge the host lipids and store them as TAG. The different regulation of the Tgs synthases in *M. tuberculosis* grown under stress conditions might reflect particular responses of *M. tuberculosis* to its environment, TAG carrying specific fatty acids, or different TAG locations (58).

The synthesis of peptidoglycan seemed to be a requisite for *M. tuberculosis*, especially in hypoxia (**Figure 5**). The genes encoding the proteins required for the addition of D-alanine, D-isoglutamate, D-alanyl- D-alanine and *meso-*diaminopimelate (MurCDEF) to the muramyl substrate to form the muramyl-pentapeptide as well as MurX and MurG performing the two last steps in the intracellular peptidoglycan biosynthesis (59) were upregulated in hypoxic *M. tuberculosis* (**Figure 5**). Surprisingly though, the gene encoding the L,D-transpeptidase LdtA, which forms the peptidoglycan 3→3 cross-links, was upregulated in all stress conditions. The peptidoglycan of exponentially growing *M. tuberculosis* is mostly composed of 3→4 cross-links formed by the D,D-transpeptidases DacB1/DacB2. The shift from a 3→4 cross-linking to a 3→3 cross-linking was observed in stationary phase *M. tuberculosis* and was linked to a response of *M. tuberculosis* switching to a dormant phenotype (60). The down-regulation of *dacB1* and *dacB2* and up-regulation of *ldtA* corroborates the hypothesis that in most stress situation, *M. tuberculosis* modifies the structure of its peptidoglycan to a 3→3 cross-linking, which may reinforce its structure and rigidity to better respond to external assaults.

### Virulence Factors

**Figure 6:**
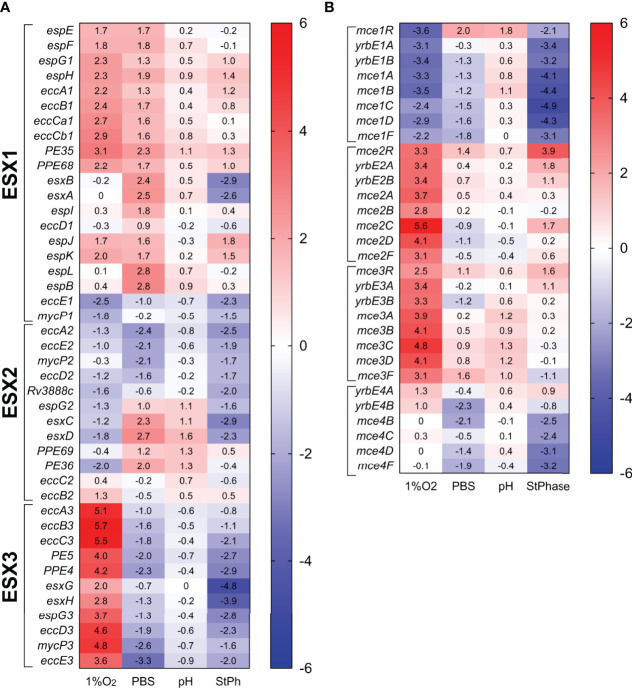
Heat map of genes expression associated with the ESX systems **(A)** and the mammalian cell entry (mce) system **(B)**. The log2 fold change for each gene and condition is indicated in the cell.

The five type VII secretion systems (ESX) of *M. tuberculosis* secrete specific components into the host and are crucial for *M. tuberculosis*’ virulence (61, 62). Deletion of part of the ESX1 system, known as region of difference 1 (RD1), is the basis of the attenuation of the BCG vaccine (63, 64). Apart from their role in the interactions between *M. tuberculosis* and its host, the ESX systems are also important for cell wall integrity and may play a role in conjugation (62, 65). The RNA-seq data set revealed that the ESX systems 4 and 5 had expression levels in all stress conditions mostly similar to exponential growth (**Table S2c**). The ESX1 system, which targets the host cell membrane, was mostly upregulated in all stress conditions in this study and a whole gene knock-out mutant exhibited no survival defect in macrophages (63). The main difference between stress conditions was the differential regulation of the genes encoding the antigens ESAT-6 and CFP-10 (EsxA, EsxB) in stationary phase and nutrient-starved *M. tuberculosis* (**Figure 6**). The ESX-2 system, also implicated in host cell permeabilization (66), was mostly down-regulated in all stress conditions, although interestingly part of the locus (*espG2-PE36*) was upregulated in nutrient starvation and acidic condition (**Figure 6**).

The most drastic difference between stress conditions was with the expression levels of the genes encompassing the ESX3 system, which were repressed in nutrient starvation or in stationary phase, and highly induced in hypoxic conditions (**Figure 6**). The ESX3 system is known to be regulated by iron and zinc and required for iron metabolism. Deletion of the *esx3* locus in *M. tuberculosis* increased the production of the cell-associated mycobactin siderophores and resulted in a growth defect in macrophages and survival in immunocompromised mice (67). While the induction of the *esx3* genes did not impact the expression of the genes involved in mycobactin biosynthesis (*mbtA-N*) in hypoxic conditions, down-regulation of the esx3 genes in the starvation or stationary phase conditions did not lead to an up-regulation of the *mbt* locus, but rather a repression of specific genes (*mbtA. mbtD*, and *mbtH*) in the mycobactin biosynthesis pathway as well as *hupB*, a gene encoding an iron-regulated protein involved in transport of mycobactins (68). Furthermore, up-regulation of *ideR* and *zur* encoding regulators of genes involved in iron and zinc metabolism was observed in these conditions (**Table S2c**). The Zur regulon is a repressor for the ESX3 system (*Rv0280-Rv0292*) (69), while IdeR negatively regulates siderophore biosynthesis but positively controls iron storage. Genes involved in iron storage such as *bfrA* and *bfrB* were not differentially regulated in any conditions, suggesting that in nutrient-starved or stationary phase conditions, *M. tuberculosis* might limit or prevent metal ions uptake and transport.

The mammalian cell entry (mce) genes had been initially associated with *M. tuberculosis* virulence since deletions of these genes affected the ability of *M. tuberculosis* to enter, grow and persist in mammalian cells (70, 71). There are four *mce* operons in *M. tuberculosis* (*mce1-4*), and each is composed of two *yrbeAB* and six *mceA-F* genes. These operons are present in pathogenic and non-pathogenic mycobacteria and have similarities with ATP-binding cassette transporters, where the Mce proteins serve as substrate-binding proteins and the YrbE proteins as permeases (72). Mce proteins participate in nutrient uptake, cholesterol uptake and catabolism (23, 73, 74), lipid homeostasis following stress responses (72) and in cell wall modification (75). Our RNA-seq data revealed that: 1) the *mce1* operon was highly repressed in hypoxic and stationary phase conditions; 2) the *mce2* operon was induced in hypoxia and moderately in stationary phase condition; 3) the *mce3* operon was only induced in hypoxia; and 4) the *mce4* operon was the least impacted showing moderate downregulation in stationary phase and in nutrient starvation (**Figure 6**). Deletion of the *mce2* operon in *M. tuberculosis* resulted in an increase in the sulfolipid SL-1 production (76). Upregulation of the *mce2* operon in our hypoxic and stationary phase experiment was associated with the down-regulation of genes involved in SL-1 biosynthesis such as *papA1, pks2* and the transporter *mmpL8*, in the same stress conditions (**Figure 5**), corroborating the relationship between the *mce2* operon expression and SL-1 biosynthesis. Others have reported that genes from the *mce1* (14) and *mce4* (77) operons were upregulated during hypoxia, which we did not observe. The Mce1 and Mce4 proteins are involved in the transport of hydrophobic substances such as fatty acids and cholesterol, respectively (74). Genes encoding proteins involved in lipid biosynthesis (Fas, Tgs, **Figure 5**) or cholesterol catabolism (**Figure S5**) were upregulated in our study validating the down-regulation of these transporters in these stress conditions. The *mce1, mce2* and *mce3* operons are known to be negatively regulated by *mce1R, mce2R* and *mce3R*, respectively (78–80). Yet, we found that in hypoxic and stationary phase conditions, the *mce1R, mce2R* and *mce3R* genes were not differentially regulated from the *mce1, mce2* and *mce3* operons. The lack of consensus between previously published studies and ours suggests that the regulation and functions of these operons could be specific to their environment.

### Genes Uniquely Differentially Regulated in Each Stress Condition

To determine which *M. tuberculosis* gene responds specifically to a growth condition and not to a general stress response, we reviewed set of genes that were differentially regulated only in one condition. Most of these genes belong to either the functional category intermediary metabolism and respiration or cell wall and cell processes (**Table S3**). Each of these stress conditions would be anticipated to be encountered upon intracellular infection, but this could vary depending on the heterogenous nature of macrophage phenotypes and indeed, the stage of infection.

### Hypoxia

Six hundred and eighty two genes were upregulated 4 to 300-fold in hypoxic *M. tuberculosis*. The most upregulated annotated genes not discussed above encoded the copper transporter CtpV (Rv0969, 300-fold) and copper-sensitive operon repressor CsoR (Rv0967, 160-fold) (81), which are located downstream of the highly upregulated *accA2/accD2* operon (*Rv0971c-Rv0976c*, see above) (**Figures 3**, **S4**), and the copper metallothionein MymT (Rv0186A, 90-fold). CsoR was predicted to be involved in *M. tuberculosis* response to hypoxia, controlling entry and exit from hypoxia (12). Furthermore, genes encoding proteins predicted to be part of *M. tuberculosis* response to copper (82) such as the potential copper regulators Rv1994c (CmtR) and Rv2642, the copper repressor RicR (Rv0190), which regulates MymT (83), the copper detoxification oxidase Rv0846c, the metal permeases Rv0849 and Rv2963, and the cadmium-inducible protein CadI were also upregulated (from 5- to 170-fold) (**Table S2c**). Notably, the sigma factor *sigC*, which was down-regulated in hypoxic *M. tuberculosis* (**Figure 2**), was shown to be required for copper uptake (84), suggesting that the up-regulation of the copper stress response regulon could be a response to the down-regulation of *sigC* in hypoxic *M. tuberculosis.* Of note though, in nutrient-starved *M. tuberculosis, sigC* was also down-regulated, but the copper stress regulon was not upregulated (**Table S2c**, **Figure S4**). This suggests that hypoxic *M. tuberculosis* aims at controlling the copper level to reduce potential copper/metal toxicity (85). In contrast, up-regulation of the ESX3 system (**Figure 6**) might hint to a specific need for iron and zinc in hypoxic *M. tuberculosis.*

Two hundred and twenty-eight genes were upregulated specifically in hypoxic *M. tuberculosis*. The upregulated pathways were in metabolism of carbohydrates (*glgBE, mutB, mqo*), sulfur (*cysQH, metA*), lipid (*fas, ppsBCD, pks13, pks5*), nucleotide (*guaB, purF, nrdB, cya*), amino acids (arginine, histidine, shikimate, the aspartokinase and dehydrogenase encoded by *ask* and *asd*), cofactors (nicotinamide, folate, vitamin B12), as well as the peptidoglycan biosynthesis (*murCDGX, pbpAB*), chaperones (*groEl, groES, dnaJ1, ftsH, mycP3*), ABC transporters from the Mce2 and Mce3 families, the ESX-1 and ESX-3 systems and two-component systems (*regX3, mtrB, cite, devR*). The most upregulated genes were *Rv0974c* (*accD2*), *Rv0972c* (*fadE12*) and *Rv0971c* (*echA17*), which are part of the *Rv0971c-Rv0976c* operon upregulated in both hypoxic and stationary phase conditions (see above) and which represent potential targets to render the tubercle bacilli incapable of surviving this key stress encountered within the host battlefield.

Four hundred and thirty four genes were down-regulated in hypoxic condition. The most-downregulated (40-fold) was *Rv0516c* encoding an anti-anti-sigma factor. Two hundred and eleven of these genes were uniquely down-regulated and were involved in gluconeogenesis (*glpX, pgmA*), energy metabolism (*frdC, ctaC, menAB*), metabolism of lipids (*tgs, hadABC*) and lipoproteins (*Rv0112-Rv0115*), as well as membrane transport (*modAB, oppBD)*, translation (*rpsN1, rpsT, rpmG2*), DNA replication and repair (*dnaE1, dnaN, dnaG, uvrD2*). The most down-regulated genes in this group *Rv3753c* (40-fold) and *Rv1115* (20-fold) were of unknown function and represent intriguing oxygen-sensitive functionalities which could be followed up using Kochs molecular postulate.

### Nutrient Starvation

Five hundred and seventeen genes were upregulated in nutrient starvation (PBS plus tyloxypol). The most upregulated were *prpD* (*Rv1130*, 420-fold) and *prpC* (*Rv1131*, 270-fold). One hundred nighty eight genes were uniquely upregulated in this condition. Among them were genes encoding proteins involved in the metabolism of nitrogen (nitrite reductases *nirBD*), lipids (polyketide synthases), in translation (30S ribosomal proteins Rpso, RpsF, RpsR2, and 50S ribosomal protein RpmG1), transcription factors (NmtR, WhiB3) two-component systems (*kdpE, mprA, glnB*). The two most annotated upregulated genes encoded the chalcone synthase *pks10* and the nitrite reductase *nirD*.

Two hundred and thirty six genes were uniquely down-regulated. More than a third of them (91) belong to the functional category of Intermediary metabolism and respiration. Among them were genes involved in: the electron transport chain (cytochrome C reductase (*qcrAC*), cytochrome oxidase (*cydB, ctaE*) and the ATP synthase *atpB*); the cell wall biosynthesis (*ddlA* (peptidoglycan), *pimA* and *Rv2611C* (lipoarabinomannan), *wbbL1*(arabinogalactan)); the metabolism of nucleotides (*purEKL, ndkA, guaA, pyrBCHR, tmk, carB*), amino acids (*leuC, argFGHJBCD, trpD, thrC*), and cofactors (NAD^+^ (*nadABC, nudC*), CoA (*ilvX, coaAE*), biotin (*bioF1, bioBD*), folate (*folCD*), porphyrin (*hemLZ, cobMLHNT*)), t-RNA biosynthesis (*gatAC, thrS, metS, proS, pheT, trpS*), and homologous recombination (*recGCD*). The most down-regulated genes were a conserved hypothetical gene *Rv0950c* (194-fold) and interestingly the resuscitation-promoting factor *rpfE* (70-fold*)*, which would be expected to rise dramatically once nutrient starvation condition would be relieved to enable the bacillus to propagate.

### Acidic Condition

The most upregulated genes among the 118 genes induced in acidic conditions were part of the gene cluster *Rv1806-Rv1808* (*ppe31-pe20-ppe32*), a set of genes of mostly unknown function, yet highly downregulated in an *M. tuberculosis* strain lacking the acid and phagosome regulated locus *aprABC*, a locus specific to *M. tuberculosis* pH adaptation in macrophage (86). This locus was also highly induced in magnesium-starved *M. tuberculosis* (87), which correlates with the upregulation of *aprABC* (12 to 45-fold) observed in our nutrient starvation experiment (**Table S2c**), suggesting that this locus may not be specific to acidic condition. Recently, one of the most prevalent PPE family of proteins *M. tuberculosis ppe31* was associated with pH sensitivity and *M. tuberculosis* survival in macrophages (88).

Only 12 genes were specifically upregulated in acidic conditions, suggesting that this may not be a major stress encountered by this intracellular survival adaptee. Two were involved in lipid biosynthesis (*pks4, papA3*) and four in cell wall and cell processes (*kdpF, iniB, kdpA, Rv3479*). *pks4* and *papA3* are required for the synthesis of di- and poly-acyltrehaloses, lipids located at the surface of *M. tuberculosis* and associated with *M. tuberculosis* virulence (89, 90). The only other annotated gene was *this* encoding a sulfur carrier protein for the synthesis of the vitamin B1 thiamine.

Among the 14 down-regulated genes, only four were annotated: the antitoxin *vapB20*, the malonyl CoA-acyl carrier protein transacylase *fabD2*, the isopentenyl pyrophosphate isomerase *idi* and PE_PGRS56. The locus *Rv1180-Rv1184c* (*pks3-pks4-papA3-mmpl10*) is associated with the production of di- and poly-acyltrehaloses, which is upregulated in acidic conditions and is also highly repressed in hypoxia and stationary phase (**Figure 5**), suggesting that the highly adaptable *M. tuberculosis* resorts to different paths and especially different lipids to ensure survival in various stress situations.

### Stationary Phase

It is highly likely that growth reduction in this manner occurs, particularly in activated macrophages. Therefore, the genes upregulated in this condition could be particularly interesting to target. The most upregulated gene in stationary phase was *Rv2662* (1300-fold), a gene of unknown function, previously found to be induced in nutrient starvation (20). This gene belongs to the common stress signature as *Rv2662* was induced in all stress condition.

One hundred and seventy seven genes were uniquely upregulated with most of them belonging to the functional category virulence, detoxification, and adaptation (mostly toxins and antitoxins) (**Table S3**). There were no clusters based on annotation that identified specific pathways. Other genes were involved in genetic information processing such as transcription factors (*smtB, whiB7*), translation factors (*infB, prfB*), chromosome (*ftsE*) and transporters (*subI, cyst, modC*).

Included in the 146 genes uniquely down-regulated were genes associated with histidine metabolism (*egtEDC*), translation (*rpsE, rplF, rplL, rplR, rpmD*), ABC transporters (*mce4, proZW, pstS1, dppC, oppA*), part of the ESX-1, -2 and -3 systems, lipid biosynthesis (5 polyketide synthases*, fadD15, tgs2*), DNA repair (*recA, hupB, recX, mutT1*) and the CRISPR-associated system (*cas1, csm2, csm3*).

### Genes Expressed in All Conditions

The analyses above were based on differential expression of genes in stress conditions compared to exponential growth. We reasoned that knowledge could be gained by examining the genes, which were highly expressed in all growth and stress conditions, i.e. correlating to the conditions most often found within in the intracellular environment.

**Figure 7:**
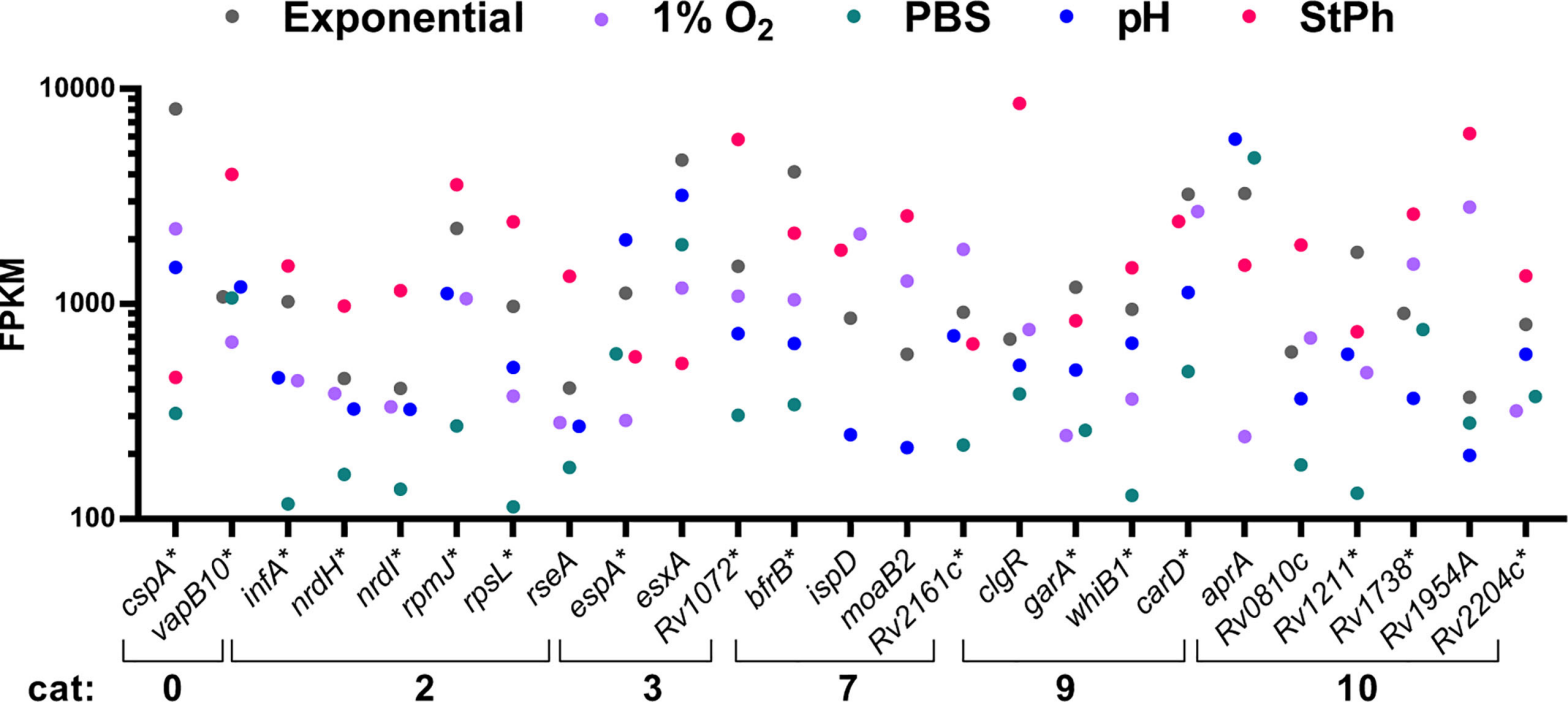
Fragment per kilobase million (FPKM) of genes in the 95^th^ percentile. The five *M. tuberculosis* growth conditions were: exponential, hypoxia (1% O_2_), nutrient starvation (PBS), acidic environment (pH) and stationary phase (StPh).

In our data set, 25 genes were among the 95^th^ percentile based on fragment per kilobase million (FPKM) in all conditions (exponential growth, nutrient starvation, stationary phase, hypoxic or acidic environment) (**Figure 7**). Of this, 68% of the genes (17, marked with * in **Figure 7**) had been previously classified in the top 100 most expressed genes in exponentially growing *M. tuberculosis* (91). Excluding the conserved hypothetical genes whose function remains to be determined, most of these genes encoded proteins involved in DNA replication (the ribonucleotide reductases NrdI and NrdH) and protein formation (translation initiation factors InFA, the 30S and 50S ribosomal proteins RpsL and RpmJ, and the anti-sigma factor sigE RseA). The Clp protease regulator ClgR, which is controlled by SigE, triggers the transcription of several proteases and chaperones essential for macrophage infection (92), and was also highly transcribed in all growth conditions. Genes encoding regulatory proteins were amongst the 95^th^ percentile such as *whiB1*, which was previously shown to be the most transcribed *whiB* gene in exponential phase (93), *garA* involved in the regulation of carbon metabolism (94) and *Rv3583c* encoding CarD, a regulator of rRNA transcription (95). The secreted effector protein EsxA along with its secretion-associated protein EspA were also highly expressed in all conditions. EsxA is part of the virulence-associated ESAT-6 secretion system ESX-1 and is highly attenuated in a murine model of infection (63).

Genes involved in maintaining bacterial growth and genome function were the most expressed genes in all the conditions tested, which was surprising considering that most of these conditions placed *M. tuberculosis* in a non-replicating state. Possible explanations would be that this illustrates the heterogeneity of growth rates within the bacterial culture or that the bacterium is continuously ready for exiting a non-replicating state and resuming a growing phase.

## Discussion

This study aimed to identify genes that were commonly expressed in the various stress conditions that the exemplar intracellular pathogen *M. tuberculosis* encounters when infecting its primary niche the host macrophage. This set of genes would define a persisting stress response that could be used to explore novel drug targets or novel antigens for vaccine development. A set of 34 genes were found upregulated in all the stress conditions tested *in vitro*. This set of 34 genes did relate to *M. tuberculosis* genes upregulated in macrophages since 26 and 27 of these genes were also upregulated in *M. tuberculosis* during infection of mouse lung alveolar and interstitial macrophages, respectively, compared to *M. tuberculosis* in liquid culture (13). Yet, the percentage of *M. tuberculosis* genes upregulated in each individual stress data set and also present in published data sets of upregulated genes of *M. tuberculosis* in mouse macrophages (13, 28, 29) did vary with the stress applied: the highest percentage was found in hypoxic and stationary phase condition (55-76%) and the lowest in acidic condition (38-45%), suggesting that the conditions where *M. tuberculosis* is in hypoxia or in stationary phase relate the best to *M. tuberculosis* in mouse macrophages.

The four stress conditions applied to *M. tuberculosis in vitro* revealed further similarities and differences between all stress conditions tested. Each of these stress conditions would be expected to be encountered within the host macrophage, but the extent may depend upon the macrophage phenotype and activation status. Indeed, *M. tuberculosis* can also survive in non-professional phagocytes such alveolar epithelial cells (63), which may not exhibit all of these hostile onslaughts. The transcriptome of the hypoxic *M. tuberculosis* had some unique differentially regulated genes consistent with the need for specific amino acids and cofactors in oxygen-deprived *M. tuberculosis*, a situation likely to be present for the majority of this bacilli’s lifecycle. Specifically, the genes involved in the biosynthesis of the amino acids arginine, histidine, methionine, the vitamin B9 folate, and the molybdopterin cofactor were upregulated. Interestingly, the transcriptomic signature of hypoxic *M. tuberculosis* was very similar to that observed in human monocyte-derived macrophages and in dendritic cells infected with *M. tuberculosis* (96). Tailleux and colleagues noted that in these host cells, *M. tuberculosis* upregulated genes associated with the *dosR* and *kstR* regulons, arginine and histidine biosynthesis, lipid degradation, cholesterol catabolism, and nitrate respiration (96), genes that were also upregulated in hypoxic *M. tuberculosis.* Together these results suggest that the response of *M. tuberculosis* to 1% oxygen may be the most relevant to the conditions *M. tuberculosis* experiences in human macrophages. Furthermore, Tallat and colleagues (97) defined an *in vivo-*expressed genomic island that included *M. tuberculosis* genes exclusively upregulated in infected mice but not in liquid cultures. Two third of the genes included in this region, *Rv0960-Rv0991*, were also upregulated in hypoxic and stationary phase *M. tuberculosis* indicating that the metabolic and environmental status of *M. tuberculosis* in mice may be very similar to these two stress conditions.

The persisting stress response encompasses a set of genes involved in generating and metabolizing propionate. One of the most upregulated loci related to propionate metabolism in the persisting stress response is *prpCD* of the methylcitrate cycle. The methylcitrate pathway processes propionyl-CoA into succinate and pyruvate to be incorporated into the TCA cycle. Propionate and its derivative propionyl-CoA are toxic metabolites generated *via* degradation of fatty acids or cholesterol, both being a primary source of nutrient and energy for *M. tuberculosis in vivo*. Although cholesterol was not present in our experiments, most of the genes associated with cholesterol degradation (98) were upregulated in the stress conditions, especially the genes associated with generating propionyl-CoA from cholesterol (*hsaFG, Rv3540* (98)) (**Figure S5**), suggestive of an adaptation triggered by these stresses to survive and persist within the intracellular environment. Furthermore, genes involved in the degradation of the amino acids valine and isoleucine (*bkdCBA, accD1, mmsA*, **Figure 3**), which yields propionyl-CoA, were also highly upregulated, indicating that, under stress, there is an influx of propionate being processed by *M. tuberculosis*. Lee et al. demonstrated that in macrophages *M. tuberculosis* detoxifies propionate/propionyl-CoA *via* two pathways: the methylcitrate pathway and the methylmalonate pathway, which uses propionyl-CoA to synthesize methyl-branched lipids such as PDIM, sulfolipids or acylated trehalose derivatives (22). Exposing *M. tuberculosis* to *in vitro* persisting stress mimicking conditions encountered in the host/macrophages yielded a similar response to what was previously described by Lee *et al*: an upregulation of genes involved in the methylcitrate pathway and the methylmalonate pathway, but also in cholesterol degradation and in the biosynthesis of methyl-branched lipids (**Figures 3**, **5D**, **S5**). The latter one was stress-specific as genes implicated in the biosynthesis of PDIMs (*ppsABCD*) were mostly upregulated in hypoxic condition, while genes associated with the biosynthesis of sulfolipids and polyacylated trehaloses (*pks3-mmpl10, Rv3822-pks2*) were upregulated in acidic and nutrient starvation conditions and down-regulated in hypoxia and stationary phase (**Figure 5**). All these elements suggest that propionate and propionyl-CoA production and metabolism play an important role in enabling *M. tuberculosis* to persist. Although *prpCD* was indeed highly upregulated in hypoxic, acidic, nutrient-starvation and stationary phase conditions and also found upregulated in *M. tuberculosis*-infected macrophages (13, 26, 29), this locus is not essential for the survival of *M. tuberculosis in vivo*. A study by Muñoz-Elías demonstrated that *prpCD* was indeed required for growth in mouse macrophages, yet deletion of *prpCD* in *M. tuberculosis* did not affect *M. tuberculosis* survival and persistence in mice (99). This illustrates the difficulties in finding new targets for drug or vaccine development.

The stresses that leave *M. tuberculosis* in a persistent state may yield important clues on how to sterilize an *M. tuberculosis* infection, but understanding the events that lead to the sterilization of *M. tuberculosis* is primordial. *M. tuberculosis* has evolved in response to an ongoing killing assault by macrophages in the mammalian host and has developed efficient resistance mechanisms to survive sterilization and persist for long periods in its mammalian host. Persistence in *M. tuberculosis* has been defined as the single greatest impediment to TB control. The goal in studying the biology of *M. tuberculosis* in persisting conditions is to find new ways to sterilize *M. tuberculosis* infections. The set of data generated in this study might offer new leads to study the intricate interplay between biological pathways and useful comparators to future studies. Ultimately, we need new approaches to sterilize *M. tuberculosis* infections to reach this ultimate goal of ending TB epidemy.

## Data Availability Statement

The datasets presented in this study can be found in online repositories. The names of the repository/repositories and accession number(s) can be found below: Gene Expression Omnibus (GEO) database, accession number GSE199263.

## Author Contributions

CV performed the stress experiments, analyzed the data and wrote the manuscript. BY performed the RNA-seq experiment, analyzed the data, reviewed and edited the manuscript. RC and SH-W analyzed the data and edited the manuscript. LE analyzed the data, reviewed and edited the manuscript. WJ planned the experiments, reviewed and edited the manuscript. All authors contributed to the article and approved the submitted version.

## Funding

WJ acknowledges funding from the National Institutes of Health grant AI26170. SH-W acknowledges the Medical Research Council for funding MR/N007328/1.

## Conflict of Interest

BY and LE are employees of New England Biolabs, a US company that sells research reagents (such as RNA reagents and library preparation kits) to the scientific community.

The remaining authors declare that the research was conducted in the absence of any commercial or financial relationships that could be construed as a potential conflict of interest.

## Publisher’s Note

All claims expressed in this article are solely those of the authors and do not necessarily represent those of their affiliated organizations, or those of the publisher, the editors and the reviewers. Any product that may be evaluated in this article, or claim that may be made by its manufacturer, is not guaranteed or endorsed by the publisher.
