# Supplementary material for: Commonalities of Mycobacterium tuberculosis Transcriptomes in Response to Defined Persisting Macrophage Stresses
Source: Front Immunol. 2022 Jul 1;13:909904. doi: 10.3389/fimmu.2022.909904 (PMC9283954; doi:10.3389/fimmu.2022.909904)
Supplement: Supplementary file 1 [file Table_1.pdf]

Table S1. Number of read pairs and percentage of rRNA for each library.

Name	Number of read pairs	bowtie2 mapping	rRNA
starvation_rep1	5,599,683	98.46%	0.30%
starvation _rep2	7,416,507	98.02%	2.46%
exponential_rep1	6,878,334	98.96%	0.25%
exponential _rep2	6,182,938	98.67%	0.13%
hypoxic_rep1	5,422,587	97.50%	0.48%
hypoxic _rep3	4,403,598	92.45%	4.23%
pH_rep1	5,820,638	98.42%	0.16%
pH_rep2	5,102,034	97.64%	1.02%
stationary_rep1	6,640,871	98.06%	0.86%
stationary_rep2	7,118,437	97.84%	0.61%
