# Supplementary material for: Commonalities of Mycobacterium tuberculosis Transcriptomes in Response to Defined Persisting Macrophage Stresses
Source: Front Immunol. 2022 Jul 1;13:909904. doi: 10.3389/fimmu.2022.909904 (PMC9283954; doi:10.3389/fimmu.2022.909904)
Supplement: Supplementary file 3 [file Table_3.pdf]

Table S3. Classification of the genes uniquely induced or repressed in *M. tuberculosis* grown under hypoxic, starvation, acidic or stationary phase condition compared to exponential growth condition. In parenthesis is given the percentage relative to the total number of genes in the *M. tuberculosis* genome in each category.

**Functional category**	**Hypoxic**		**Starvation**		**Acidic**		**Stationary phase**	
log2 fold change	> 2	< -2	> 2	< -2	> 2	< -2	> 2	< -2
virulence, detoxification, adaptation	27 (12)	10 (4)	3 (1)	15 (6)	0 (0)	1 (0)	24 (10)	7 (3)
lipid metabolism	30 (12)	10 (4)	12 (5)	16 (7)	2 (1)	1 (0)	5 (2)	15 (6)
information pathways	10 (4)	14 (6)	10 (4)	17 (7)	0 (0)	1 (0)	4 (2)	10 (4)
cell wall and cell processes	55 (7)	52 (7)	43 (6)	36 (5)	4 (1)	2 (0)	20 (3)	40 (5)
insertion seqs and phages	11 (7)	3 (2)	3 (2)	2 (1)	0 (0)	3 (2)	12 (8)	2 (1)
PE/PPE	22 (13)	1 (1)	8 (5)	2 (1)	1 (1)	1 (1)	9 (5)	5 (3)
intermediary metabolism and respiration	80 (9)	37 (4)	24 (3)	91 (10)	3 (0)	1 (0)	17 (2)	25 (3)
regulatory proteins	14 (7)	6 (3)	16 (8)	8 (4)	0 (0)	0 (0)	16 (8)	5 (3)
conserved hypotheticals	39 (4)	76 (7)	78 (7)	49 (4)	2 (0)	4 (0)	66 (6)	36 (3)
