# Supplementary material for: Commonalities of Mycobacterium tuberculosis Transcriptomes in Response to Defined Persisting Macrophage Stresses
Source: Front Immunol. 2022 Jul 1;13:909904. doi: 10.3389/fimmu.2022.909904 (PMC9283954; doi:10.3389/fimmu.2022.909904)
Supplement: Supplementary file 4 [file DataSheet_1.pdf]

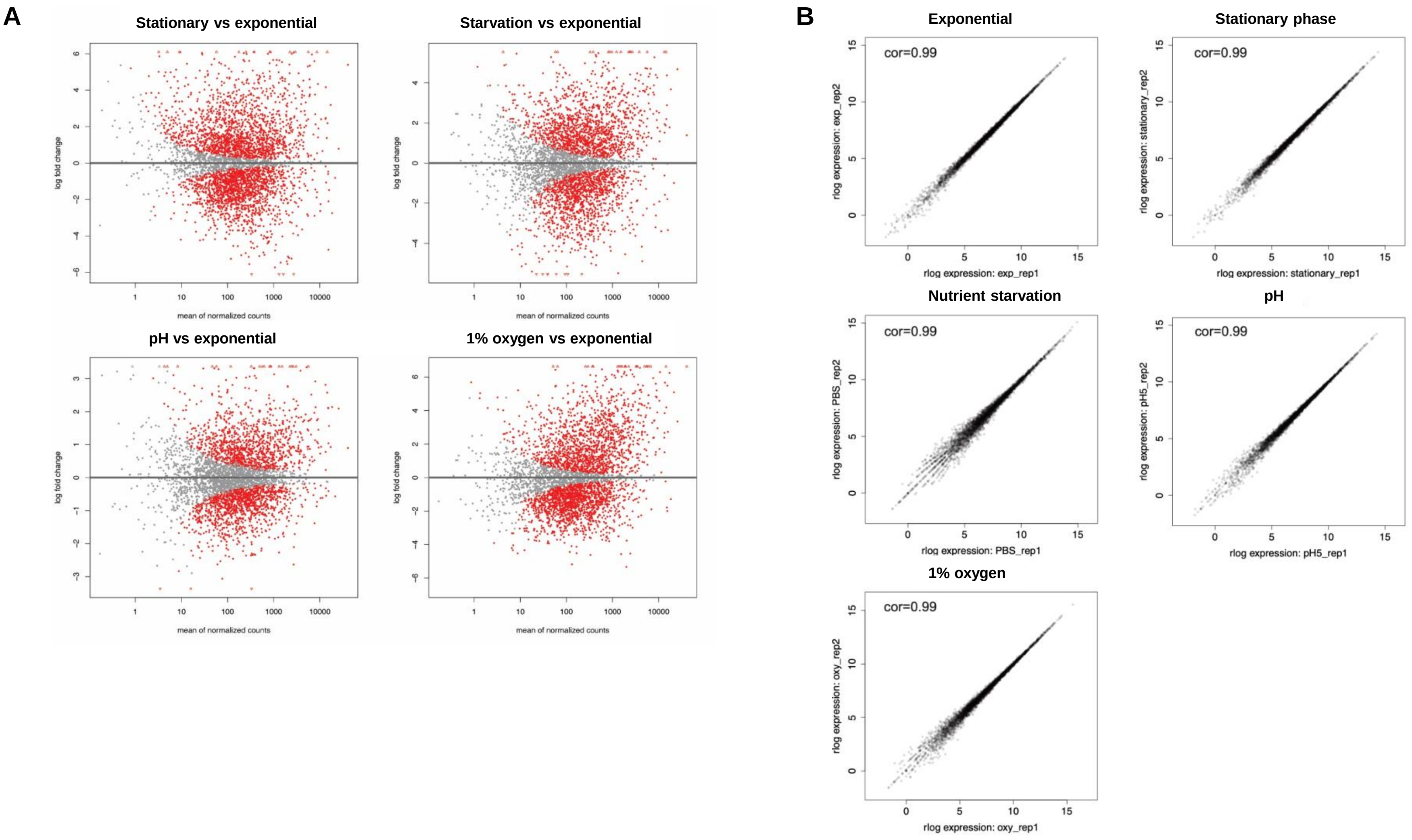

Figure S1: (**A**) The MA-plot shows the log2 fold changes (stress condition compared to exponential condition) over the mean of normalized counts. Differentially expressed genes with an adjusted *p* value below 0.05 are shown in red. (**B**) r-log correlation of the replicates.

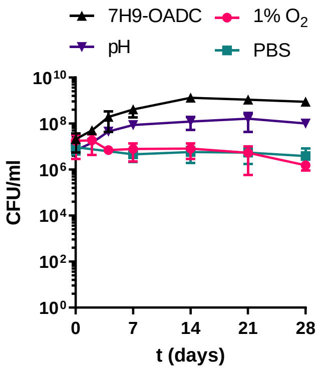

Figure S2: Viability of *M. tuberculosis* H37Rv grown in rich media (7H9-OADC), in hypoxia (1% O_2), in acidic media (pH) or in nutrient starvation (PBS). At the indicated times, samples were removed, serially diluted and plated to determine CFU/ml. Mean with SEM is plotted (n = 3).

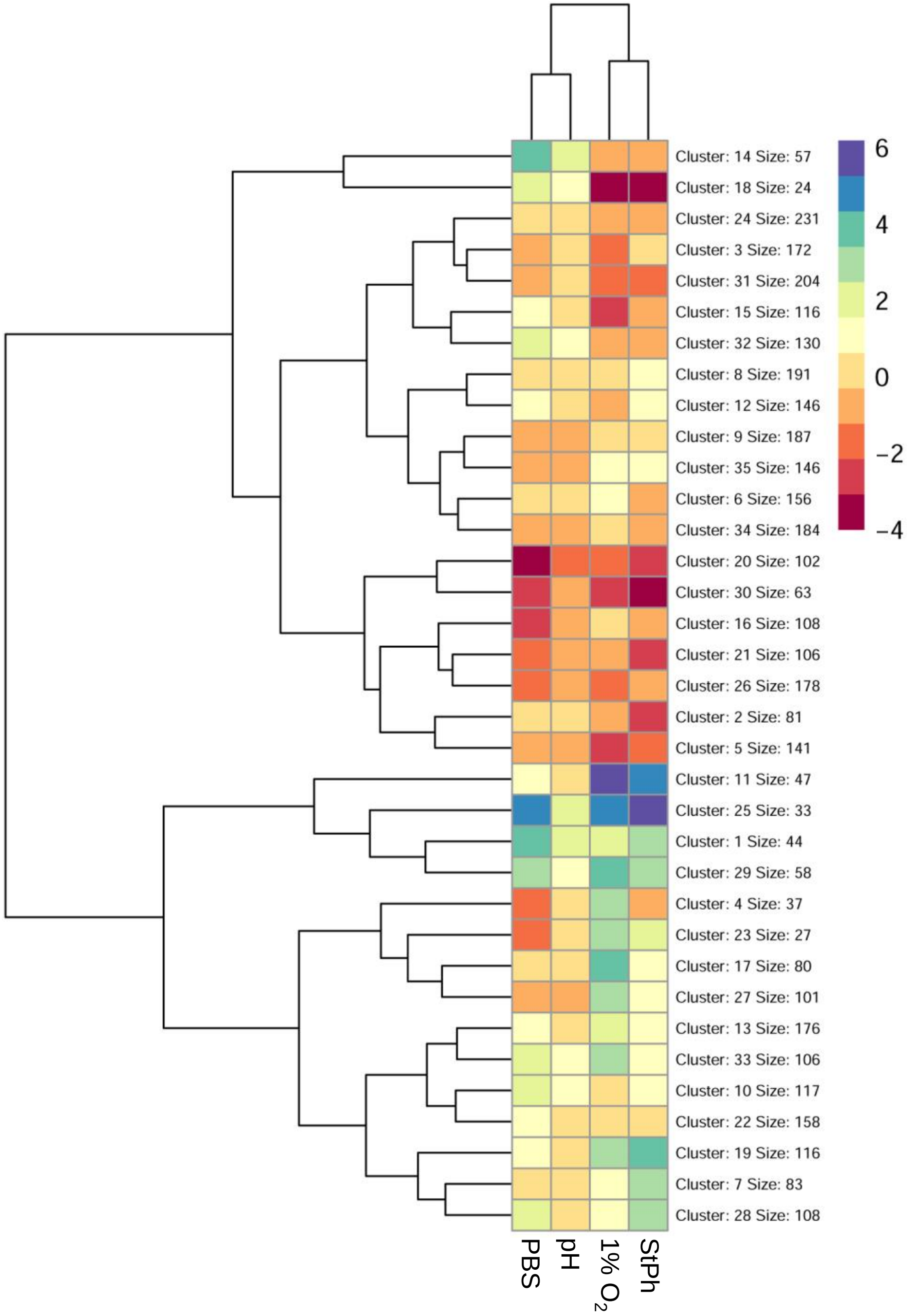

Figure S3. Heatmap of the *k*-means clustering with k = 35. The list of genes included in each expression cluster can be found in Table S2.

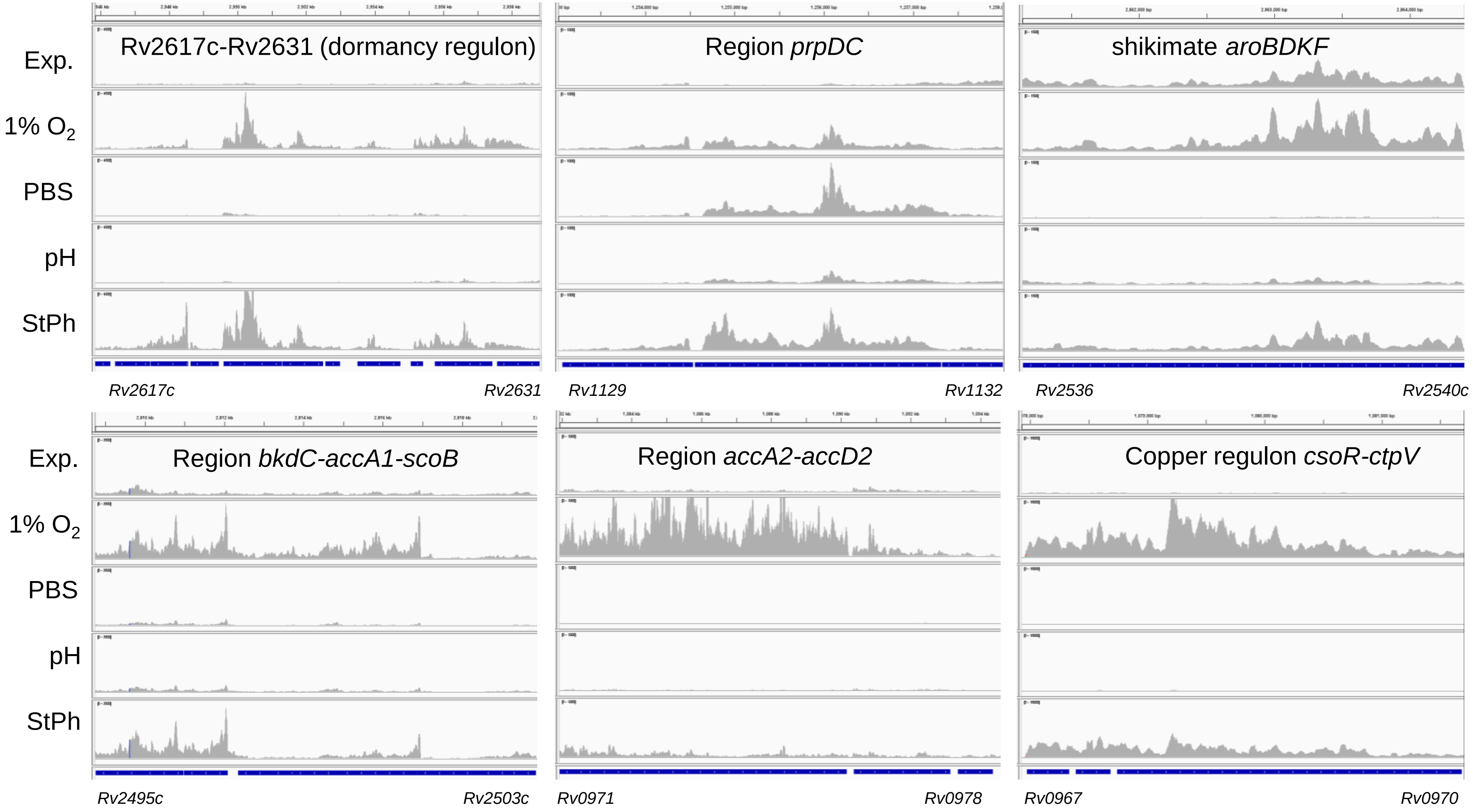

Figure S4. Sequence alignment of specific *M. tuberculosis* regions in the five growth conditions. The five conditions were: exponential growth (Exp.), hypoxic (1% O_2), nutrient starvation (PBS), acidic media (pH), and stationary phase (StPh). The genes encompassing each region is indicated at the bottom of each graph. The same scale was applied in the sequence alignment of the five growth conditions for each individual region.

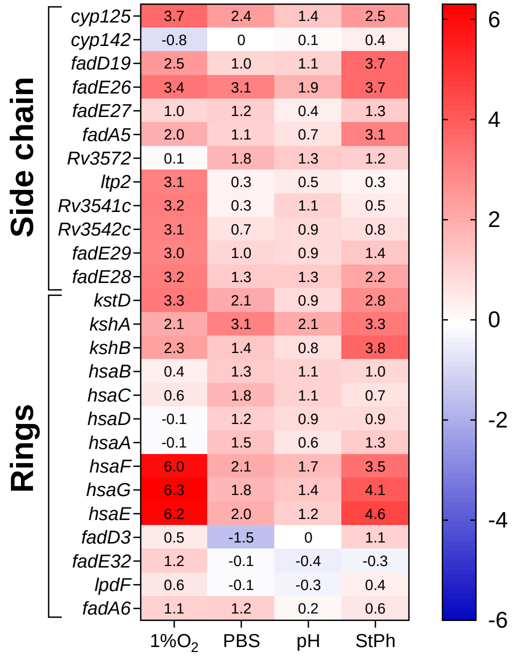

Figure S5. Heat map of genes involved in cholesterol degradation. The log2 fold change for each gene and condition is indicated in the cell.
